# Healing Multiculturalism: Middle-Ground Liberal Forgiveness in a Diverse Public Realm

**DOI:** 10.1007/s11406-016-9758-z

**Published:** 2016-10-11

**Authors:** Monica Mookherjee

**Affiliations:** SPIRE, Keele University, Staffordshire, ST5 5BG UK

**Keywords:** Political forgiveness, Liberalism, Multiculturalism, ‘love of the world’, Empathy, Compassion

## Abstract

This article examines debates about political forgiveness in liberal, pluralist societies. Although the concept of forgiveness is not usually taken up by liberals, I outline a plausible conception by exploring two recent approaches. The first, ‘unattached articulation’, concept requires no real emotional change on the forgiver’s part, but rather a form of civic restraint. In contrast, the second version highlights a strong form of empathy for perpetrators. In spite of their advantages, each concept proves too extreme. The problems are revealed by focusing on the case of the Harkis, who fought for the French during the Algerian war. Often still marginalised in French society, their case helps to highlight the conceivability of a ‘middle-ground’ or moderate concept of political forgiveness. Its core rests on the forgiver’s care for the social world. While this concept brings considerable challenges also, and is not inevitable in any particular case, it entails a more plausible combination of emotional and rational shifts in the forgiver’s world-view. Although the article does not recommend forgiveness by any person or group, it observes, recalling Arendt’s idea of *amor mundi* or ‘love of the world’, that political forgiveness may sustain a viable connection between diverse citizens’ public and non-public lives.

## Introduction

Debates about forgiveness and reconciliation abound in culturally-diverse societies like South Africa, Australia and Northern Ireland, which seek to move beyond bitter histories of group conflict. While few political philosophers would reject the value of reconciliation, liberal multiculturalists might question the political goal of forgiveness. One worry is that calling on members of wounded groups to forgive will fail to lead to reconciliation. In reality, it may only support self-regarding, grudging compromises which fail to secure anyone respect. A different liberal concern is that to forgive the perpetrators of mass injustice would demand too much. The requirement to change one’s emotional attitude towards wrongdoers, even when they repent, might seem to equate to exoneration. The act of forgiving may, from this perspective, undermine liberal values of retributive justice and reciprocity in a diverse state.

While my aim in this article is ultimately to defend the possibility of a form of liberal political forgiveness, the above concerns relate to two versions of this political goal which would be equally problematic from the perspective of liberal multiculturalism. I shall call the first version the ‘unattached articulation’ concept of forgiveness, and the second the ‘change of heart’ approach. By exploring the difficulties with each, this article puts forward a third possibility. The middle-ground concept proposed would prove potentially preferable and more plausible, I suggest, to citizens in a diverse liberal public realm.

I develop this approach by focusing on the case of the Harkis, who fought as auxiliaries (*supplétifs)* in the French-Algerian war which ended in 1962. Now a highly diverse and creative community in France today, their troubling wartime and post-war experience highlights the demanding nature of political forgiveness for many minority groups. Part of the challenge confronted by the Harkis in addressing the past may be that, by fighting with the French, they were taken to have ‘betrayed’ their fellow Algerians, even if the term ‘betrayal’ scarcely seems to represent their motives and actions. On the other hand, the Harkis themselves were not well supported by the French after the war’s end. Highlighting their predicament in his wide-ranging and nuanced analysis, *The Harkis: The Wound that Never Heals,* Vincent Crapanzano (2011: 176–77) at one point suggests the possibility that perhaps today the ‘solution’ for the modern Harkis lies in forgiveness, even though it would involve transcending a seemingly impossible emotional threshold.^1^ Yet, in presenting a fascinating, impassioned and empathetic portrait of this minority, Crapanzano is aware that the idea of forgiveness in this context calls for a discussion of the effects of decolonization and the politicisation of memory in a liberal-republican regime (see Sung 2012). Crapanzano’s suggestion is therefore intriguing. It invites a considered discussion, including but not only in relation to a diverse society such as France, of the meaning and conditions of forgiveness in a political sense.

It is worth being aware from the beginning of the problems with presuming that political philosophers may offer concrete views on whether forgiveness should apply in a particular case. This article therefore does not recommend forgiveness for specific persons or groups; it only aims to unfold what it offers as a potentially plausible form of the concept, drawing on the Harkis example largely to examine the advantages and challenges that this concept brings. Accordingly, the discussion is structured in the following way: the next section (section 2) outlines some initial concerns about political forgiveness from a liberal multicultural perspective. This is followed, in section 3, by an outline of the first concept, namely the ‘unattached articulation’ version. I suggest that this concept underestimates the cost to Harki and other wounded minorities of having to ‘bracket’ their deepest grievances in the public sphere. Section 4 then considers the polar opposite, namely the ‘change of heart’, view, based on the possibility of empathy. Suggesting that this conception proves equally demanding of wounded individuals, I explain that the close identification assumed between the perpetrator and the transgressor is likely to be implausible after deep group conflicts. The final section develops the ‘middle-ground’ concept based on compassion for the social world. It presents a mid-point between full empathy and the studied indifference in the two versions previously. Involving a combination of moderate, though still in some cases existentially challenging, rational and emotional changes, this diversity-centred concept recalls Arendt’s (1958) concepts of natality (newness), and *amor mundi* or ‘love of the world’.

I concede that this form of forgiveness will not always be possible, and it may not achieve the closely connected goal of reconciliation. Yet, it offers a potentially productive middle-ground between condoning injustice too quickly, on the one hand, and, on the other, compromising without any real change of mind. Seeming humanly attainable and socially sustainable, then, this mid-way approach avoids ambitious calls for empathy, and rests instead on the possibility of developing compassion for the social world. It recalls Arendt’s idea of ‘love of the world’, or a mature social acceptance of the impossibility of forever binding others and oneself to the past.^2^ While wounded individuals should not be called on to deny their moral judgements, this form of forgiveness fosters agency in the context of diversity, and promises to break a cycle of alienation. Although it offers no guarantee, in the Harkis’ case or in any other, the idea of forgiveness outlined in this paper could assist to open a pathway to reconciliation, and is thus potentially restorative and political, for the right reasons and in the right way.

## Liberal Forgiveness in a Diverse Public Realm: Unity, Divergence and the Harkis

Liberal theorists have not devoted as much attention to forgiveness as to other virtues like civic respect and reciprocity. One reason for this is that forgiveness might appear a-political, and therefore irrelevant to the public sphere. However, it is worth beginning with the contrary view of at least one prominent political philosopher. In her classic *The Human Condition* (1958), Hannah Arendt initially places the idea of forgiveness outside the worldly ethic of the political sphere. Forgiveness in its original, Christianised sense, she argues, is a-political*,* belonging to morality rather than to politics (1958: 239). In politics, she explains, we might confront the unforgivable, as famously portrayed in *Eichmann in Jerusalem* (1963). Confronted with the possibility of evil, the compromises of political affairs are unsuited to those who are truly good, whose virtue takes the form of a ‘radical innocence’ and a purity that is corrupted by politics (1958: 241).

Despite this, Arendt contends that forgiveness can, and actually must, enter politics too. This is because it supports the capacity for ‘natality’, or the creation of newness, which is intrinsic to human freedom, the utmost political concept. Forgiveness is the only reaction in human affairs that, through the respect it evinces for the common world, retains the original quality of action. It not only re-acts but ‘acts anew and unexpectedly, unconditioned by the act that provoked it’ (1958: 241). Forgiving ‘serves to undo the deeds of the past, whose “sins” hang like Damocles’ sword over every new generation’ (1958: 243). It is the only response that comprehends the frailty of the world and the capacity to create newness from this unpredictability. Without political forgiveness, past actions would appear ‘irreversible’, to a point that all future action and therefore politics would be suppressed (1948: 243).^3^ In fact, controversies exist over whether Arendt’s concept finally depends on an unworldly form of Christian love (*agape),* which would be difficult to translate into a secular political idea (Honig 1993; Bernauer 1987). Without having the space in this article to discuss this question fully, I suggest only that for many readers Arendt’s concept is fully secular and independent of Christian theology. It focuses on a political form of respect, which requires an emotional distance between citizens rather than the intimacy of Christian love (Arendt 1958: 242; Digeser 2001: 17).

Liberal multiculturalists may be unpersuaded by Arendt’s concept, however. On one side, it is a response to what Arendt calls ‘human plurality’ (1958: 6–7), a concept which holds that each person has a unique perspective on life and the world. Evaluating this perspective requires the presence of others, and this is why, as Aristotle held too, politics is essentially ‘pluralist’. While a conception of forgiveness grounded in human plurality would appeal to liberals, Arendt’s republican ideal seems fully to politicise the act of forgiveness, leaving little to the private sphere. In this sense, liberals might have reservations (c.f. Flatham 2005: 73). Moreover, Arendt’s reflections on forgiveness seem too abstract to fully explain how modern, complex states composed of different cultural or religious groups would encourage this virtue. The concern may be that secular political forgiveness, at least as portrayed by Arendt, might involve unacceptable intrusion into the psychological life of individuals. The dissolution that it seems to require of the public-private boundary seems hard to justify from a liberal perspective (Philpott 2006). Even if liberals do at times encourage emotional reactions such as civic pride (Watkins 2015),^4^ which might involve shaping private attitudes, the defence of political forgiveness might still seem to dissolve, and not only traverse, the public-private border (Bhargava 2000).

This problem may be highlighted by introducing the challenging case already alluded to in the Introduction. In this paper, I focus on the Harkis, who fought as auxiliary troops for the French (*supplétifs)* in the Algerian war. Whilst siding with French colonizers against their native Algerians, many Harkis retain their Arab Muslim identity through the trauma that they encountered after the war. Regarded as ‘traitors’ by their fellow Algerians, many were killed in massacres by the Algerians after the war. About 85,000 Harkis (derived from *harka*, an Arabic term for ‘military movement’) who survived were resettled in impoverished camps on the peripheries of French cities.^5^ Marginalised by the French, they were equally spurned by Algerians for their ‘betrayal’, even though this term may not meaningfully describe their motives and actions. Lacking a homeland, the situation of the Harkis remains precarious. As Enjelvin and Korac-Kakabadse (2012): 161) explain: ‘the pragmatic measures which were implemented by successive French governments, and which were a mixture of paternalism and indifference, resulted in the Harkis who arrived in France being assigned to the periphery of […] French identity’. Although public efforts were recently made to recognise their wartime contribution, and while Jacques Chirac spoke openly of the bravery of the troops (De Quetteville 2001), full social inclusion the Harkis, and their distinct Arab-French identity, remains elusive.

With this background in place, we can return to the possibility mentioned earlier by Vincent Crapanzano that perhaps forgiveness offers a ‘solution’ for this complex and dynamic minority community today (2011: 176–7). This view might seem intuitively problematic. One concern might be that forgiveness given by a state, if this is what ‘political forgiveness’ means, would involve institutions taking a partisan view of the past which would fail to be neutral between different interpretations of history. For instance, some French citizens view the acts during and after the war as unfortunate, best forgotten, but justified (Silverstein 2012). In their view, ultimately there would be nothing to forgive. From the perspective of many Algerians, the Harkis were ‘traitors’. Yet, as it frequently seems that terms ‘traitor’ and ‘betrayal’ are very complex to apply in this case, any institutional view would perhaps involve judgements which are too controversial. If, in contrast, the idea is that the Harkis themselves forgive, let us say, the French state, there seem to be many questions immediately about whether such forgiveness would have to depend on a public apology or a truth commission, neither of which has occurred.^6^

In fact, these questions indicate just the beginning of the controversy about political forgiveness. In particular, the relationship between forgiveness and apology is a difficult issue, and one to which we will return. For now, let us consider another crucial question confronting the concept regarding its place and role in a diverse society. Specifically, could the concept of political forgiveness resonate with different world-views and with different religious or metaphysical perspectives? If the concept rests on a culturally dominant conception of the good life, such as the Christian story of a fallen humanity, the concern may be that the commitment to public forgiveness would be seen as imposed, rather than legitimised and endorsed by members of different communities.

To respond to this concern, it is worth noticing first that the French republican focus on non-domination, unity and neutrality (*laïcité*)^7^ have surely made it harder politically for the Harkis to express a public identity based on their cultural and religious norms. To raise this point is not to suggest that the historical harms experienced by minorities must be expressed through their own cultural values. Certain human experiences transcend the boundaries of any world-view, and ought to be expressible in impartial public reason.^8^ The Harkis’ grievances could be expressed in the neutral public language of, for instance, ‘displacement’, ‘social exclusion’ and ‘marginalisation’. While this seems possible, the concern is that the potential response, in terms of political forgiveness, cannot also be neutrally or impartially justified. To be more specific, would the defence of political forgiveness assume what cannot be reasonably counted on, namely that all citizens accept a thick cultural or religious narrative, such as the Christian story of a fallen mankind?

We can further appreciate the importance of this question by understanding that many in the contemporary Harki community remain, in Crapanzano’s view at least, caught in a ‘haunting silence’ (2014: 198). While, as we shall later find in Crapanzano’s ethnography that this portrait of silence and despair is in fact more complex, the point helps us to see that collective memories and perceptions of victimhood are often experienced in group-based, culturally-grounded, terms. For this reason, it seems important that public reasoning and policies resonate with local beliefs and values, which provide languages in which human experiences make sense. If public forgiveness is a potential, or even ‘the only’, solution for members of this community, liberal multiculturalists might ask if the value presumes an account of the good life which appears ‘too Christian’ from any alternative religious point of view (Crapanzano 2014).

In this context, Auerbach (2005) aptly suggests that, if different groups with their own conceptions of ‘final’ matters such as redemption and the afterlife share convictions regarding the nature of forgiveness, religion may contribute positively to justifying this value politically. However, world-views conceive the nature and conditions of forgiveness differently.^9^ For example, despite its emphasis on confession before God, Christian forgiveness is usually unconditional, and understood as the interpersonal equivalent of love. This concept was famously exemplified by Pope John Paul II, who, on surviving an assassination attempt, forgave his assailant unconditionally (McCullough et al. 1997). In doing so, he drew on Christ’s willingness to suffer for humanity (Gregory Jones 1995; Philpott 2006: 276). In contrast, other Abrahamic religions such as Judaism and Islam make forgiveness extremely conditional. Forgiveness in Islam (*ghufrana*) is allied to demanding processes of *tawha* (repentance), which are linked to their Jewish equivalent *teshuvah* (Auerbach 2005: 480). Islam encourages forgiveness between believers, but does not recommend it in regards to infidels or apostates.

Even if forgiveness is to this extent an essentially contested concept, it could be that different cultures and religions will overlap on its core meaning in such a way that legitimates the value politically. For instance, in endorsing the policy of the acclaimed South African Truth and Reconciliation Commission, Desmond Tutu suggests such an overlap in *No Future without Forgiveness* (1994). In this text, he recommends a form of forgiveness justified by both Catholicism and the *Ubuntu* theology of African tribes (Tutu 1994; Haws 2009). Some political theorists are, however, unconvinced by this ‘overlapping’ defence of political forgiveness, not least because they worry that religion typically offers a conception of personal rather than political forgiveness. Shriver (1995: 7) observes that for religious citizens love translates into forgiveness in the public sphere; but this might prove too demanding for many ordinary, self-interested people. Conceivably, too, the unconditional demand to ‘turn the other cheek’ could undermine the basic religious beliefs of some. This point leads to a more general observation about the tense relation between religious theologies and forgiveness. Whilst religious institutions have provided resources for conflict transformation in divided societies (Philpott 2006), they have also justified violence in the recent history of countries such as Northern Ireland and Rwanda. Turning instead to a secular political concept of forgiveness, Shriver observes that: ‘if forgiveness is to escape its religious captivity and enter the ranks of ordinary definition, it has to acquire a more precise, dynamic and politically contexted definition’ (1995:7).

This seems to be an important point. For, while Arendt observes that Jesus first discovered the role of forgiveness in human affairs (1958: 238), she also saw no reason ‘to take it any less seriously in a strictly secular sense’.^10^ The unconditionality of some religious forms of forgiveness, then, and their appeal to a divinity as the ultimate authority, contrasts with secular versions which appear more realistic, and conditional on interpersonal reparations and apologies. While we will return presently to other aspects of Arendt’s thought, my aim is to consider firstly some of the neutral, secular political conceptions of forgiveness emerging after Arendt (e.g. Shriver 1995; 2003; Elshtain 2003; Digeser 2001). Their neutrality seems apt in our central case of the Harkis, given their settlement in republican France and the history of the nation’s church-state separation. Political forgiveness may need to be consistent with different religious world-views in a minimal way, but depending ultimately on religious theologies may be divisive. With these considerations in mind, I now consider one particular neutral, secular conception which might help to unfold the meaning of Crapanzano’s intriguing, though very demanding, idea.

## Liberal Multiculturalists at one Extreme: the ‘Unattached Articulation’ Conception of Political Forgiveness

A helpful way to begin to compare different forms of secular forgiveness might be by asking what the concept essentially involves. Forgiveness, it seems, always involves the forgiver’s emotional, psychological or at least behavioural change in some form. Although this is not all that forgiveness essentially involves, this point seems to provide a meaningful foundation. This focus, moreover, seems confirmed by the definition of forgiveness as ‘the intentional and voluntary process by which a victim undergoes a *change in feeling or attitude regarding an offence*, lets go of negative emotions such as vengefulness, with an increased ability to wish the offender well’ (American Psychological Association 2006, emphasis added).^11^ Although this definition is derived from the psychological literature, in much philosophy and ethics too forgiveness is taken to involve a change in emotion and attitude by the victim away from bitterness and resentment (e.g. Hampton 1988; for further references, see Digeser 2001: 16, note 5).

Starting with this general definition, we can introduce our first, emotionally modest, conception, one which seems attractive to secular liberals at first glance. Agnostic concerning the private world-view of the victim and forgiver, it does not anticipate a full overcoming of grievances or resentments. It seems persuasive in conditions of deep diversity, because, while it is not always so, past grievances may deeply inform the identity of members of some minority religious and cultural groups, in ways that are challenging fully to set aside or overcome.

A compelling example of this form of public forgiveness has been developed by Peter Digeser (2001). Although Digeser does not relate his idea to the condition of cultural pluralism, the changed attitude that he recommends seems apt. It involves only a public willingness to continue with others civically, irrespective of the sentiments or motivations that one might hold in the private or cultural sphere, and notwithstanding personal grievances.^12^ Digeser’s view respects the fact that, in conflicts ranging from Israel-Palestine to the Northern Irish Troubles, past harms are continually regarded as justified by each side. In this conception, therefore, victims could remain aggrieved in the non-public sphere of life, where group identities sometimes form through a shared memory of injustice in such a way that maintains the identity or dignity of the community (Brudholm 2008). The essence of Digeser’s conception of forgiveness is only a form of self-disclosure, to use Oakeshott’s term, or what J.L. Austin’s calls an illocution. It refers to a verbal or ‘attitudinal’ indication by the forgiver of the civic status of the other. As such, in Digeser’s words, the conception ‘swings free of any particular sentiment’ (2001: 17).

This conception is attractive mostly because of its emotional modesty. In fact, it may be the most that can be expected in conditions of cultural pluralism. Digeser, for his part, is sceptical of sentiment-based approaches to political forgiveness partly because, if they are grounded in what he calls, following Oakeshott again, ‘self-enactment’, or the ability to work on one’s deep or private feelings and transform them, there would be no reason for forgiveness to be political*.* The act of forgiving by renouncing resentment would be a purely mental act (Digeser 2001: 22). Moreover, because resentment need not result in any negative act, or any act at all, and since the sentiments underlying forgiveness can be various, conflicting and not fully transparent even to the agent, Digeser’s conception is open-minded with respect to the reasons that motivate it. This is not to say that its independence makes it arbitrary or irrational. Digeser observes that people may have various instrumental reasons for forgiving: public forgiveness may enhance a reputation for generosity; it may serve as an expression of concern; more often, perhaps, it may further one’s desire to move on with life; or, one may forgive because forgiveness is believed to further the cause of peace (2001: 12). The relevant change is only temporary disengagement from any contrary sentiments in the non-public sphere. This is all that the conception requires, because:

‘[i]n [this] conception, politics is more a matter of civic behaviour rather than appropriate sentiment. The plausibility of this conception of politics turns on the difficulty of truly knowing the feelings that motivate an action. […] What lurks in the heart of the individual may never be adequately brought to the surface’ (Digeser 2001: 17).

Let us call this conception of forgiveness the ‘unattached articulation’ concept. It involves only a public disclosure of the willingness to forgive, accompanied by a release of the other from future acts of vengeance or retaliation. Developing Digeser’s concept, it may be that, after violent conflicts, people would probably not forgive in this way in a sudden event or as a matter of a momentary decision. Rather, it seems more likely for embattled communities such as the Harkis to come about in time after particular overtures and reparations by others. It seems realistic to assume that people often will not wholly overcome negative emotions, but that they might eventually restrain themselves from allowing their negative sentiments to interfere with forming or continuing civic relations. Digeser further explains his perspective as follows:

‘By detaching political forgiveness from resentment and understanding it as an act of self-disclosure, the public character of political forgiveness is illuminated […]. I postulate that a party must convey the message of forgiveness with the appropriate standing […] Something must not only be said or somehow conveyed, but what is said must have the effect of releasing the debtor […] from the debt’ (2001: 28).

For Digeser, the articulation or behaviour that conveys the term ‘I forgive you’ need not be unconditional. It could be based on some rectificatory justice,^13^ which renders it reasonable and potentially realistic in our example. It could be that the recognition of the Harkis’ bravery, for instance, accompanied with concrete social measures to assist their situation, would sufficiently motivate at least some members of this group to forgive politically.^14^ The approach might be consistent with the Harkis’ world-view, and seems to appreciate the power-relations between this minority and the dominant society. The point is important because a strong requirement to ‘let go’ of the past completely might be oppressive to some communities, whose identity may be invested in complex ways in their understanding of the past as involving a violation. Far from demanding consensus on reasons for forgiveness, this approach respects citizens’ private sphere and does not presume to know their reasons for forgiving. This also makes forgiveness seem humanly attainable rather than a very ambitious emotional or spiritual task. Digeser emphasises that this concept cannot replace other values in political life such as rectificatory justice. But, he rightly emphasises that sometimes political forgiveness could play a role too, if only by enabling civic life to continue (2001: 18; see also MacLachlan, 2012).

Although this conception seems initially attractive, and akin to forms of public pardon in democratic states (2001: 201), it also brings certain problems from a multicultural perspective. While it appeals to the idea of reasonable negotiation between diverse people, its psychological remoteness seems, oddly enough, too demanding. It may be one issue for an entity such as a state to release a wrongdoer from future debts. However, to encourage victims of past injustice to react in this way publicly, even after some rectificatory justice, might risk failing to redeem the past sufficiently (Bennett 2003). Perhaps unexpectedly, it might operate as one-to-one forgiveness between friends.^15^ However, it is an ambitious demand in public life, in which the role of forgiveness would most likely be to help to build reconciliation between social groups. To suggest a potentially dramatic switch between public and non-public emotions may underestimate the cost that maintaining such a large ‘gap’ might involve. However contradictory it may seem, it is possible that for some Harkis, an identification with their grievance, or their ‘wounded attachment’ in Brown’s (1995) sense, may be the condition of their public sense of self.

Moreover, the deeper concern is that this concept is not a genuine form of forgiveness, or at least not of a kind that would take the parties convincingly towards reconciliation. Sceptics might point out that Digeser’s idea could be theoretically consistent with self-alienation and perhaps unforgiveness itself, concealing a social and political disaffection which people might then express in different ways. Relatedly, Nietzsche famously argues that it is the narcissistic reaction of the wounded that leads them to seek ‘public’ strategies such as forgiveness, in order to soothe the pain of past harms. Whilst, for Nietzsche, resentment is a sour and life-denying reaction, forgiveness in his understanding is not so much an antidote as a psychological tool which foments the bitterness that it hides (Nietzsche 1956[1887]). While this may seem a rather dramatic implication to draw from Digeser’s idea, Martha Nussbaum (2014) has, not dissimilarly, asked whether forgiveness is always a genuine reversal of anger.^16^ While these concerns about ‘purely public’ displays of forgiveness are perhaps extreme, they also do not seem completely groundless. The worries, furthermore, are that this conception of political forgiveness might not only fail to lead to real reconciliation, but that it can also risk foreclosing efforts to secure equality. Depending on the nature of the dispute, Ure seems right to warn that equal recognition seems ruled out, if one party publicly claims moral superiority to forgive (2007: 66)*.*

This point is concerning from a liberal perspective. While the capacity for restraint is generally crucial to the functioning of a diverse public realm, Digeser’s concept perhaps tends to seem like tolerance or even expedience. Moreover, although in Enright’s famous psychological framework for interpersonal forgiveness (2001), the process is often taken to begin with a self-regarding motive (for instance, with a recognition of the disadvantages of failing to forgive, or of how forgiveness enables health), to confuse this kind of motive with forgiveness as such fails to account for the hard psychological work involved. As we shall see, there seems to be a need, in real forgiveness, to reframe the other’s acts and integrate the meaning of the past within one’s inner life or non-public world-view. Because it seems to disconnect the public world of justice from the Harkis’ ‘private’ world view, Digeser seems to disregard the risk of losing identity that forgiveness might involve for wounded minorities (Crapanzano 2011: 156).

One the one hand, then, this concept seemed initially attractive in its appeal to Shklar *Faces of Injustice* (1990), and its appreciation of the non-ideal nature of the world. It does not assume that human beings can be better than they are, but aims to take people ‘as it finds them’ (Digeser 2001: 13). Perhaps ultimately, however, the problem lies in *not* taking people as they are, or not taking them as *people.* The concept seems more appropriate in cases where a state, or another third party institution, relieves a debtor, such as in bankruptcy cases (MacLachlan, 2012). While Digeser seems right to aim for a secular concept, ‘unfreighted with the theological baggage’, as he says, of Christianised forgiveness, the risk is created of only deepening existing injuries.

## Liberal Multiculturalism at another Extreme: Political Forgiveness as a ‘Change of Heart’

The difficulties inherent in the concept above prompt us to a second version, one which, in contrast, acknowledges the large emotive task that forgiving politically may involve after ethno-cultural conflicts. While I shall steer away from depicting political forgiveness as an ‘extraordinary’ gift, as Jacques Derrida (2001) has portrayed it, I shall now consider another formulation which also requires deep alteration of the forgiver’s world-view, but one which might more realistically suggest the challenges and prospects for forgiving politically in conditions of deep social and cultural diversity.

Taking my cue from Cheshire Calhoun’s well known article (1992), I call this conception the ‘change of heart’ approach. As we saw, forgiveness is usually understood to involve a shift in attitude or behaviour in the hope of reconstructing healthier relations (Murphy 1988). The change envisaged by this conception may or may not involve condoning the wrong and exonerating the wrongdoer; and it may or may not be supplemented with retributive justice. But, for Calhoun, it would involve being able to tell a biographical, if not a moral, story about how the other committed the wrong, attempting, with St. Augustine, to ‘hate the sin but love the sinner’. If this approach seems initially compelling, in this section I shall consider the attractions and the drawbacks of this concept from a liberal multicultural perspective..^17^

More specifically, this approach envisages the potential forgiver’s mental change towards a form of empathy, which is ordinarily defined as the ability ‘to understand and share the feelings of another’,^18^ and thus assumes a very close identification between the forgiver and the forgiven. Arguably, empathy connotes a certain ‘oneness’ or synonymity between people (Schwartz 2002). Although it may seem preferable to the emotional distance of the previous approach, and although there are stronger and weaker conceptions of empathy, the concept risks too greatly blurring the boundaries between self and other and between the public and the non-public. For these reasons, I shall suggest that this version of political forgiveness will probably be too ambitious in our central case, and, therefore, equally difficult to justify in liberal public reason.

In order to unfold this argument carefully, let us consider an example of this approach, namely Donald Shriver’s (1995) *An Ethic for Enemies: Forgiveness in Politics*. Shriver advances four components of his concept of political forgiveness: (a) moral judgement/consensus on truth; (b) forbearance from revenge; (c) empathy with the wrongdoers; and (d) hope for the construction of the relationship. The first condition involving a consensus on the truth seems contestable, as political forgiveness often seems necessary in the wake of bitter ethno-religious conflicts precisely where the interpretation of history is disputed or full consensus is hard to find. Yet Shriver’s other three conditions of political forgiveness seem firmly interlinked and consistent. Pivotal to this concept seems to be condition (c), namely the possibility of empathy.

In order to understand Shriver’s conception of empathy, we might ask whether he assumes so close an identification between the parties that the boundaries between them become dissolved.^19^ As Schwartz (2002) outlines, it is possible that empathy intrinsically involves a certain blurring of the borderlines between one’s own interest and those of the other. Although Coplan (2011) does not view it as central to empathy as such, she is worried about the risks of such a loss of differentiation between self and other through ‘imaginative identification’, which she takes to possibly undermine empathy by leading to ‘false personalisation’ of the other’s perspective and to personal distress.

To evaluate whether these problems follow from Shriver’s approach, consider his reasons for focusing on empathy as the core of forgiveness. Only by learning to place ourselves in the other’s shoes, or by trying to identify with their experience of being human, can we respond to atrocities on a global scale. That is, only the *re-*humanization of others can fully counter the de-humanization that often occurs during conflict situations and beyond. Shriver astutely explains that it is owing to a failure of empathy that violence on such a massive scale is often justified (Shriver 1995: 123–125). Exemplified acutely by relations between the United States and Japan in recent world history, such bitter conflicts arise because of a profound alienation of self and other, which is not easily overcome.

As such, Shriver is fully aware of the challenge that empathy presents: ‘empathic understanding coupled with moral horror’, he explains, ‘is a difficult intellectual-emotional achievement’ (1995: 130–131). War often depends on a calculated denial the other’s humanity. The enemy is depicted as ‘remote, monolithic, a different species’. For Shriver, the wilful ignorance of the other generated by fear of difference explains much of the harm that strangers inflict on one another, a harm which is perpetuated by refusal to conceptualise the other as human. However, this refusal is only made possible by romanticising one’s own existence or forgetting one’s history. The amnesia involved in the American dehumanization of the Japanese, and vice versa, was perhaps understandable but ultimately hypocritical, Shriver contends, because:

‘It is easier to pin the label *atrocity* on the excesses of the enemy while finding excuse for the excesses of the people that one counts one’s own. “Our atrocities” are episodic, committed in the heat of human extremity; “their atrocities” are unceasing, despicable […] My Lai is our exception; Nanjing is their rule […]. The limits of empathy can soon be reached by anyone who attempts exercises such as this: not only is it difficult to understand how one’s fellow humans act in the midst of circumstances that one has never encountered personally; but the mind does not, at some point, *want* to understand’ (Shriver 1995: 132; emphasis in the original).

Shriver’s point seems very persuasive. However, one concern might be that empathy would not lead to political forgiveness in the wake of the most violent conflicts. Different communities may differ in regards to when they would view empathy as appropriate. Arguably, too, empathy is a much stronger emotional response than is absolutely needed for basic rights to be respected and civil life to continue between individuals and groups, as liberal realists would hold (Shklar, 1990; Digeser 2001).

Shriver is aware of these problems. Yet, to establish why empathy is possible, and thus the key that enables human beings to transcend the dehumanization involved in events like Pearl Harbour and Hiroshima, he introduces a further value on which he seems to believe all parties to a conflict would agree, namely value of *life* (1995: 126). This is despite the fact that the movement from empathy to forgiveness on the basis of ‘life’ as a cardinal value may beg many questions. Members of violated groups might reasonably withhold public forgiveness on account of the life-denying actions of the perpetrators of mass injustice. The Harkis, for instance, might choose not to forgive on grounds that their empathy can extend to the perpetrators of certain acts during war itself, but not to the perpetrators of the post-war massacres. In addition, ‘thick’ conceptions of the value of life (e.g. the Christian belief that all are equally children of God, or the Buddhist belief in the pervasiveness of suffering) may support different understandings of humanity, and, thus, of the objects of empathy. There is for this reason often a complex link between reason and emotion for most people in deciding when it is reasonable and justifiable to forgive. The cultural and religious reasons offered by different world-views on the value of life may not the lead to consensus on the circumstances of empathy. This, in turn, suggests that the values of empathy and life would not generate consensus on political forgiveness in specific cases.

While empathy and life may well be respected by citizens of most major cultural or religious world-views (e.g. Eisenberg 2002), empathy can have different specific meanings and these meanings might lead to different normative conclusions. To relate the issue to our central case, for the Harkis the call to build on private empathy to forgive politically might appear mysterious, or even to add insult to their injuries. In fact, as Crapanzano (2011) is aware, after the Harkis’ post-war experiences, the horror of which many bore in silence according to the Islamic principle of *sabr* (constance; resoluteness), forgiveness may seem simply impossible. Even if some people who have experienced atrocity may over time seek to understand the others as they understood themselves, people often do not necessarily have the moral energy to decide to act on this renewed understanding. This is so, even if they have a rational and cognitive understanding of the other’s actions, which may (arguably) culminate in a certain form of empathy. Within the terminology of the psychoanalytical debate about empathy, people may have the ability for ‘empathy-as-identification’ but not necessarily for active ‘empathy-as-care’ (see Eklund 2013).

This idea leads us to perhaps the most compelling concern about empathic political forgiveness from a political philosopher’s perspective. The grounding of political forgiveness on empathy may undermine the Harkis’ legitimate non-public or ‘cultural’ sphere, by presupposing too much identification between selves. Rather, crucial to maintain from the liberal viewpoint may be the essential separateness and distinctiveness of persons. By seeming to blur, if not dissolve, this separateness of persons, empathic political forgiveness seems to undermine the crucial element of forgiveness as a secular political concept, namely that it is the object of human choice based on critical judgement between different people about its reasonable conditions. If different people cannot make critical judgements, rationally and emotionally, political forgiveness would seem a ‘foregone conclusion’ or inevitability, rather than a meaningful social choice.

Significantly, Amstutz (2007: 563) raises a related concern with respect to the empathic concept by observing that Shriver curiously does not ‘confront the inherent tension between justice and forgiveness, punishment and reconciliation’. As such, the approach seems to say little about the deliberation needed for citizens to assess the past and present critically, and to assess the conditions of justice that must occur if one is to fully be ‘moved’ by empathy to forgive the other politically. Of course, one could in a very basic sense empathise with others simply by recognising them as sentient human beings. This might motivate a one-to-one personal forgiveness for very simple transgressions. However, this basic form of empathy, which somewhat dissolves the separateness of persons, seems plausible only where there are no complex moral concerns, human rights violations or ‘crimes against humanity’ in question. Thus, a basic sense of empathy for the humanity of the other could understandably ground forgiveness in rather unchallenging or morally simple situations. But it is unlikely to support a political concept of forgiveness in ethnically diverse situations, as the latter must involve some element of critical evaluation and choice between distinct people, often with different views of the world.

Therefore, while Enright and Fitzgibbons (2000: 79) seem right to believe that forgiveness must generally involve a certain reframing, which enables the person who has suffered to re-think the situation and to see the other as a ‘person who is, in fact, a human being, and not evil incarnate’, public forgiveness still calls on different emotional resources in the forgiver. These emotional resources have less to do with recognising the other as a human being in a generic sense, and more to do with critical deliberation and judgement about persons with a potentially different world-view.

In summary: I have suggested that a strong notion of empathy threatens to dissolve the distinction between self and other necessary for the publicity and conditionality of political forgiveness. It also, relatedly, weakens the distinction between the non-public (private or ‘cultural’) and public spheres. Even if the change of heart towards Shriver’s strong notion of empathy may assist the path to interpersonal forgiveness, this concept does not easily translate into a political form of the concept. In conditions of social diversity, people have individual subjectivities which they may opt, or not opt, to express in political ways (Crapanzano 2011). The private empathy of one individual towards another might conflict with the group’s equally important desire to retain their ‘wounded attachments’ (Brown, 1995) as a condition of preserving identity in their struggle for recognition in a diverse state.^20^ Private empathy with another’s basic humanity could well coexist with continuing to disagree with the perpetrators on their moral evaluation of the past and, thus, with withholding public forgiveness. Briefly, therefore, I have suggested that it is doubtful that political forgiveness across diverse communities could, or should, require citizens to feel what others feel, to assume or internalise their predicament as their own, or, in the more extreme definitions of empathy, ‘make less distinct the differences between self and other’ (Hodges and Klein 2001).

The difficulties with the two conceptions of public forgiveness considered so far point to the need to form a middle-ground between the ‘unattached articulation’ and ‘change of heart’ approaches. This seems apt because a number of recent conceptions, such as Montiel’s (2002) idea of ‘socio-political forgiveness’ and Andrews’ (2000) ‘negotiated forgiveness’, also observe the need to locate this complex idea between the polarities of alienating diverse citizens from the values and sentiments of their private sphere, and assuming ambitiously that personal one-to-one reactions can fully politicised. Thus, the notion of liberal political forgiveness brings particular challenges. If symbolic, identity-related conflicts cannot always fully be resolved through familiar appeals to rectificatory or redistributive justice (Waldron 1992), the challenge seems to be to define a form of public forgiveness which would not be too ‘unattached’ and yet not so ‘attached’ as to dissolve the boundaries between selves.

## Healing Multiculturalism: the Harkis, *amor mundi* and Compassionate Forgiveness in a Diverse Public Realm

I am now able, at this stage in the article, to build on the insights of the previous concepts to outline what I hope to be a middle-ground, or moderate, conception of political forgiveness. The core of this concept is found in a special kind of care for the social world, which is supported by Arendt’s concepts of natality, as mentioned previously, and *amor mundi,* or ‘love of the world’ (1958). These concepts in turn sustain Arendt’s concept of forgiveness, and her political thought as a whole. Although Arendt resisted liberal politics and went beyond the liberal focus on civic restraint and political freedoms (McCarthy, 2012), her idea of love of the world through the receptivity to newness (‘natality’) supports the rational and emotional shifts which I shall suggest to be central to political forgiveness in a liberal-multicultural sense.^21^ To emphasise again, my goal is not concretely to recommend that the Harkis or any other specific people forgive. Rather, it is to define an emotionally plausible conception, with the awareness that it would ultimately be a moral and political choice.

The conception to be outlined draws a more viable link than the previous versions between a person’s non-public identity and their public sentiments. This link enables us to avoid the motivational problems with two concepts considered earlier. The conception of ‘compassionate’ political forgiveness does not assume the intimacy of empathy or the complete emotional distancing of the ‘unattached articulation’ approach. The middle-way that it advocates is important in the light of Watkins’ (2015: 29) suggestion that public forgiveness need not presuppose consensus between the parties on a moral judgement about the past. People might see the value of continuing future relations out of a form of civic respect, rather than by judging transgressors favourably and redeeming them. For, as we have seen, such resistance to redemption may be central to the identity and world-view of victims.

In spite of this, compassion for the predicament that all would face if forgiveness were absent from the world might motivate the wronged to consider that the other may possess different but reasonable interpretations of history. Through this recognition, victims might come to exhibit what Arendt calls ‘love of the world’. Acutely aware of the vulnerability the world, according to Bernauer (2012) Arendt views humans as only fully functioning as such when they understand that ‘worldliness’, the awareness of oneself as an element of a larger whole, provides a more attractive form of life than the commodification and obsession with individuality characteristic of modernity (Arendt 1958: 248). By this, Arendt meant to defend acting on the basis of human plurality and an awareness of diversity from its detractors. The capacity to act for the common world provides, for Arendt, an antidote to the ‘alienation of the world’ not only exemplified in crimes against humanity, most horrendously in the concentration camps, but also perpetuated by modernity’s preoccupation with personal subjectivity and the individual’s inner will (Bernauer, 2012: 3).

Earlier in this article, I suggested that Arendt’s concept of political forgiveness does not fully elaborate the link between the forgiver’s sentiments in the public and non-public spheres. However, crucial to a viable liberal-multicultural conception of forgiveness does seem to be her underlying ethic of worldliness, or an orientation of openness to the world which forms of totalitarianism and human rights violations typically deny (Arendt 1958: 248). Keeping Arendt’s ethic in mind enables us to conceptualise political forgiveness as involving modest, but still in some cases challenging, rational and emotional shifts. Cognitively, people would need to ‘decide’ that forgiveness is a sensible and rational option from the perspective of their own private sphere. At the same time, for real forgiveness it seems necessary to accompany the rational change with an emotional shift in the public sphere. As I will explain, this emotional change would most likely need to involve at least some ‘heart-softening’ towards the transgressor, even if not a full reversal of negative emotion or a perfect mental connection of the empathic approach.^22^

Although it is very difficult to specify the precise combination of shifts that would be necessary and sufficient for particular cases, I shall at least outline some examples to illustrate what these changes could involve. The rational change could include coming to see that forgiveness might improve one’s health or decrease emotional stress; that it might build self-esteem and that of others in the group; that it might inspire future generations; or that ‘moving on’ with life is pragmatically necessary (Enright and Fitzgibbons, 2000: Haws, 2009). However, this rational aspect would need to be accompanied by an emotive change, probably a dissipation of rancour and increase of positive mental energy. Through this, the person would focus outwards on the world rather than ruminating constantly about past events, however strong their moral objection continues to be (Haaken 2002). Again, my purpose is not to prescribe but only to suggest that at least a limited emotional shift may come about by conceptualising, for instance, the transgressor as a person who acted under their own specific pressures or psychological ‘failings’. Alternatively, greater emotional intelligence might come about through recognising that the other was responding to a set of human challenges and circumstances, or that, from their standpoint, there might have been no ‘right answer’. Political forgiveness, then, might involve the imaginative awareness that no alternative might have been obviously better, all things considered, from that person’s point of view. This is not to say that the person was simply following an order, or, literally or figuratively, that they could not have acted otherwise. Rather, it is the emotional awareness that the goods and bads flowing from different options could have weighed almost evenly from the other’s point of view.

Taken as a whole, this approach to political forgiveness seems supported by recent recognition of the need to weaken the reason-emotion distinction in philosophical definitions of forgiveness. On this view, people have more ‘rational optionality’ concerning the emotions defining forgiveness than is commonly believed (Allais 2013: 637). I do not suggest that this form of public forgiveness would be easy by any means for victims of severe human rights violations, but only that it seems more attainable than the previous conceptions across the boundaries of communities in ethically-diverse states. Clearly, the difficulty that it presents would vary in proportion to specific people’s experience of injustice. It could be that it would simply not be conceivable for a people that has experienced genocide, for instance, to begin to make such emotional and rational changes towards political forgiveness.

If it is at least conceivable in cases less extreme, or in one sense more complex, than extensive genocide, however, care must still be taken in regards to what public forgiveness of this sort may accomplish. If it is not accompanied by the commitment by all to work for relations of non-domination, forgiveness will probably not lead to reconciliation.^23^ Also, this conception of forgiveness seems most likely, and perhaps only possible, after reparations or a public apology. While space does not allow me to explore the relation between concepts of forgiveness, apology and reconciliation, it is very likely that the three concepts are closely tied, with political reconciliation possibly being the ultimate point or goal of forgiveness itself.^24^ This form of forgiveness is, therefore, highly conditional and deliberative. In this conception, to forgive politically is to retain a sense of oneself as a distinct person, as separate from the other. It is not to dissolve the separateness of persons, or to ‘move on’ with the belief that the past did not occur.

Could this middle-ground conception of forgiveness apply realistically in our central case? Any suggestion regarding the Harkis’ forgiveness must be made with care, as Crapanzano himself became acutely aware in his wide-ranging dialogues with them:

‘I suggested […] in my conversations with the more sensitive Harki children that the only way they could be liberated from the burden they bore as Harkis was by pardoning the French. However, as they knew and I knew, this was impossible for they had no platform from which to proclaim forgiveness. What were they to do? Stand up in front of the Elysée and say, *La France, je vous pardonne?* One woman said that she had thought about this but realised it was impossible […] And other Harkis could make no sense of what I was saying. *Forgiveness was simply impossible’* (Crapanzano 2014: 199, emphasis added).

While the voices above draw attention to the deep sense of powerlessness experienced by some members of this group, their sentiment of ‘having no platform’, Crapanzano’s engagements with this complex minority also remind us of the risk of generalising the experience of powerlessness, and, perhaps, too, of failing to recognise the vital issue that power is invariably accompanied with resistance. Part of Crapanzano’s point is to view this minority as internally diverse (2011: Chapter 1), with subjectivities that cannot be straightforwardly generalised. If he is correct to resist hasty conclusions about a singular Harki consciousness or identity, or about the consciousness or identity of any human being, then it seems that the form of forgiveness suggested in this article may not be in all cases impossible. The diversity of literary works, including drama and novels, from second and third generation Harkis in France testify to the internal complexity of their individual subjectivities. These subjectivities, in turn, seem to suggest that different political responses are possible (Reeck, 2006; Ireland, 2010).

It is at least conceivable, then, that some members of deeply injured groups come critically to assess for themselves to what extent maintaining a sense of victimhood has configured their individual and collective lives. They might assess, rationally, how this might have been an excuse for inaction, or a source of resentment that cannot be resolved without forming the emotional resources to move forward. In the aftermath of social conflicts, when material needs have been met, I have suggested that their forgiveness might involve the ability to care for the context, the ontological condition, in which future action is possible. Through this concern, citizens may maintain a realistic connection between their public and non-public spheres, without dissolving the boundary between them.

There may be concerns, however, that beginning this process would be too arduous. How could those who have been deeply violated and maltreated even start to think about compassion for the social world in general? I emphasise that it is possibly not an option which all would be able to consider. But it seems, despite that, a legitimate and plausible social choice for some. Importantly, the object of ‘compassion’ in this conception would not be the specific transgressors, such as the French military officials in our current example. In fact, these officials, even if they were to apologise, would perhaps always risk being ‘tarnished by the wrongdoing they have committed’ in the eyes of many Harkis (Crapanzano 2014: 204). Yet, in view of the life-denying dilemma that all would experience if forgiveness were absent from the world, I suggest that the object of compassion would be an entity outside the particular relationship between self and other that produced the original conflict. While such forgiveness may be sometimes impossible, its possibility in others is hinted at by the eloquent voices that younger Harkis have brought to their communities (Enjelvin & Korac-Kakabadse, 2012). Should they opt for public forgiveness, the process would involve the key to forgiveness: the refusal to pass pain back to the transgressor or to others (Enright and Fitzgibbons 2000: 79).

Although this form of political forgiveness may be a possible step in the road to reconciliation, it is obviously arduous and hardly a foregone conclusion after deep ethno-cultural conflicts. Minimally, it requires the ability to look outwards from the self, and this may only be begun when one’s basic identity is not threatened. Human beings require a sense of belonging, self-esteem and consistency as the basis from which further future action might proceed (Max-Neef 1991). Political forgiveness, I believe, is precisely not a foregone conclusion but a matter of critical judgement in the particular case. Yet, it could be that such a reaction is precisely the one most needed to enable a future, ‘natality’ in Arendt’s sense, which connotes to begin, to start something new, to refuse to return pain, as an antidote to the worldlessness and depersonalisation of past violations.^25^ Finally, care for natality arises from the awareness that no one can forgive themselves. It arises from the recognition that we risk the future, the creation of which is the special task of humans, by thinking that public forgiveness is impossible.

## Conclusion

This article has contended that, in spite of the controversies surrounding it, public forgiveness may be a legitimate and plausible option in a diverse liberal public realm. I explained, however, that victims of historical injustices are unlikely to forgive by empathising with their transgressors. While private empathy is undoubtedly a very significant human capacity, citizens are more likely to forgive publicly through care for social world, or through an ethic of worldliness in Arendt’s sense, cultivated through a series of (apparently) moderate, those in some cases still challenging, emotional and rational shifts. While it is no minor attainment for victims of serious injustices, compassion-based forgiveness would link the cultural and political spheres of identity more plausibly than the ‘change of heart’ and ‘unattached articulation’ concepts.

By occupying a more persuasive middle-ground, this conception responds to the two liberal concerns about political forgiveness noted at the outset. To recall, the first was that in the real world political forgiveness often looks like a grudging compromise, which fails to redeem the past or heal any wounds. The second was that political forgiveness would prove too demanding after bitter ethnic conflicts. It would require a full identification with the other and renunciation of resentment, with the potential loss of group identity that such psychological acts might entail.

In the end, political forgiveness cannot be imposed or prescribed. It is only one option which may or may not assist reconciliation. I have referred to the creative and dynamic Harki communities in France today to demonstrate the challenges involved in political forgiveness, without the idea that they, as individuals or as a community, should forgive. In spite, then, of the inevitable open-endedness of my discussion, its purpose has been to draw attention to the need in liberal-multicultural political morality at least to consider the relevance of concepts such as forgiveness, which have often appeared alien to theories more at home with rights, procedures and rules. The form of public forgiveness suggested resonates with Arendt’s view that it is, at times, only by forgiving that diverse people may emerge strongly from past troubles, with a capacity to love natality and to act anew in and for the world.

## Footnotes

1 The question of who should forgive whom in the case of the Harkis may be seen from one perspective as not straightforward, however. There seems to be a set of complex moral relationships in this case. In this article, the question considered is whether the Harkis might forgive the French. Admittedly, this is a controversial and ambiguous question, which raises the further dilemma of whether the recipient of forgiveness would be the wartime or the contemporary state.

2 Arendt is more typically taken to view love as ‘anti-political’: ‘love, by its very nature is unworldly, and it is for this reason rather than its rarity that it is not only apolitical but anti-political, perhaps the most powerful of all antipolitical human forces’ (1958: 242). However, my suggestion in this chapter is that notion of a political form of love, which is love for the idea of beginning, newness or *natality,* may be read as Arendt’s specific recuperation of Aristotle’s concept of *philia politike.*

3 As one referee for *Philosophia* has suggested, the ideas of irreversibility and action in Arendt’s thought are deeply relevant to her conception of political forgiveness. Arendt’s understanding of ‘irreversibility’ configures her understanding of political forgiveness in particular. However, because the paper does not, as such, set out to defend Arendt’s specific version of political forgiveness, and owing to the constraints of space, a more detailed discussion of these concepts in Arendt’s thought is not possible to undertake in this article.

4 Watkins (2015) argues that the promotion of forgiveness is neither illiberal nor partisan. In contrast with the view that forgiveness would either promote a sectarian conception of the good, or would rest on a stable inference by the government of which party wronged whom, Watkins responds that the promotion of a forgiving attitude does not involve determinate judgements, and it need not even involve the wrongdoer accepting the negative judgement that seems to be implied in forgiveness.

5 The literature on the situation of the Harkis in France is extensive. Amongst the most instructive texts, see: Eldridge (2010), Evans (2002) and MacCormack (2008), in addition to Crapanzano’s study, which provides the point of focus for this article.

6 See Dominique Mois, ‘Analysis: France must find the strength to say sorry for Algerian past’, *The Scotsman,* Monday 24th October (2011).

7 Mookherjee (2016) notes reasons for discussing the French response to ethno-cultural diversity within a liberal framework, whilst observing that differences do exist between liberal and republican theories of justice. Newer republican theories such as that of Cecile Laborde attempt to accommodate religious and cultural diversity in such a way that renders it consistent, even if it still differs from, liberal multicultural theories.

8 I am grateful to an anonymous reviewer for *Philosophia* for raising these objections.

9 As Auerbach explains, a certain ‘unconditional’ forgiveness is one of the cornerstones of Christian theology, which is linked with the notion of ‘original sin’. Since all human beings are conceived in sin, and therefore doomed to a sinful life, they are entitled to forgiveness.

10 While Arendt argued that Jesus of Nazareth was the first to ‘discover’ the role of forgiveness, and did so in a religious context, even Jesus did not wish to argue that only God has the power to forgive, or that humans only do so in the name of the Father. As she writes: ‘It is decisive in our context that Jesus maintains against the “scribes and Pharisees” first that it is not true that only God has be power to forgive, and second that this power does not derive from God […] but on the contrary, must be *mobilised by men towards each-other before they can hope to be forgiven by God also’* (Arendt 1958:214).

11 Admittedly, this working definition of forgiveness may seem controversial. Many other definitions have been proposed in the philosophical and psychological literature. A common starting-point is often taken to be Bishop Butler’s seminal analysis of forgiveness in his sermon ‘Upon Forgiveness of Injuries’ (1897), in which he defines forgiveness as ‘the overcoming, on moral grounds, intense negative attitudes – the vindictive passions of resentment, anger, hatred and the desire for revenge’. This definition is taken up later by Murphy (1988). In contrast, while Griswold (2007) agrees up to a point with Butler’s definition, he does not think that this overcoming should only be a ‘moderation’, but a ‘complete elimination’ of resentment for morally creditable reasons. My reason for appealing instead to a psychological definition, is that the notion of a ‘morally creditable reason’ might be inherently controversial. However, as we shall see, while it is best to provisionally to adopt a working definition that does not appeal explicitly to a moral reason to forgive, I ultimately argue that the moral rationale for political forgiveness surfaces in the justification that we provide. It lies in forgiveness for the sake of the social contract, or for the world.

12 One might ask here whether foregoing punishment is entailed by this concept of political forgiveness. There is surely a complex relationship between the two concepts. For Digeser (2001), moreover, as we shall see political forgiveness presupposes a certain amount of ‘rectificatory justice’, which could, in principle, include punishment. The question of punishment, however, is a large issue in itself and is not the central concern of this paper.

13 For Digeser, political forgiveness requires only the following: ‘(1) the existence of a relationship between at least two parties; (2) a debt owed by one party; (3) which is relieved by a party with appropriate standing; (4) conveying the appropriate signs and utterances (5) whose success does not depend on the emotion or internal states of the forgivers’ (2001: 35).

14 As noted earlier, another condition of public forgiveness might be thought to be a state apology. However, as Crapanzano observes, it is not clear to what extent forgiveness must be conditional on a public apology, as the latter is fraught with its own difficulties concerning the sincerity and efficacy of the apology, where the parties hold different conceptions of history. He concludes that the issue of whether an apology would motivate public forgiveness ‘is speculative because France has not apologised and the Harkis have not forgiven’ (2013: 206).

15 One might, for instance, forgive simply for one’s own sake, for the sake of one’s wellbeing and health, which are supposedly compromised by excessive feelings of resentment and rancour (see Van Oyen et al. 2001).

16 I agree with an anonymous referee for *Philosophia* that a very important issue arises as to when forgiveness is genuine or sincere. I admit that, in this paper, I have not been able fully to address this question. I agree that it poses a large question, perhaps particularly where there is a level of equality between two sides to a dispute. At the same time, when one is dealing with the forgiveness of a historically violated group, and there seems to be a very large disparity of power, it seems to me that the main issue to consider in this context is perhaps not going to be whether their forgiveness is genuine. This is partly because it would often be such a large task for the violated minority to begin the process of forgiveness that it might seem odd and perhaps misplaced, in liberal political morality, to ask whether group members genuinely forgive. On the other hand, the issue of feelings in the non-public sphere is important, in the sense that forgiveness should not simply conceal anger or bitterness, as in the Nietzschean conception; and one should not merely grudgingly pretend to forgive in public. These are, in fact, amongst the problems with the ‘unattached’ conception of political forgiveness. I am grateful to the anonymous referee for emphasising this point.

17 As Arendt writes: ‘ Respect, not unlike Aristotelian *philia politike,* is a kind of “friendship” without intimacy or closeness; it is regard for the person from the distance which the space of the world puts before us, and this regard is independent of qualities which we may admire or of achievements which we may highly esteem. Respect … because it concerns only the person, is quite sufficient to prompt forgiving of what a person did, *for the sake of the person’.* (Arendt 1958: 243, emphasis added).

18 This definition is cited from the on-line Oxford English Dictionary.

19 ‘Empathy’ has many different definitions that encompass a broad range of emotional states, including caring for others, experiencing emotions that match the other person’s emotions and discerning what the other person is thinking and feeling (see Schwartz 2002). To differentiate empathy from the broader notion of care, Shriver does seem to believe that it involves an actual identification of the self with the other.

20 As Brown (1995) explains regarding the politics of resentment of wounded group identities: ‘…In its attempt to displace its suffering, identity structured by *ressentiment* at the same time becomes invested in its own subjection. This investment lies not only in its discovery of a site of blame for its own hurt will, not only in its acquisition of recognition through is history of subjection (a recognition predicated on injury, now righteously revalued), but also in the satisfactions of revenge, which they ceaselessly reenact […] *Identity structured by this ethos becomes deeply invested in its own impotence*, even while it seeks to assuage the pain of its powerlessness through its vengeful moralizing, through its wide distribution of suffering , through its reproach of power as such’ (Brown 1995: 70, emphasis added).

21 Schaap (2003) offers thorough insight into the way Arendtian values of ‘worldliness’ and ‘natality’ may be taken up in a contemporary conception of the political forgiveness. He does not, however, specifically relate his examination to liberal multiculturalism, nor recommend the combination of rational-emotive shifts suggested in this article.

22 I am grateful to an anonymous reader for *Philosophia* for raising this possibility in regards to the ‘middle ground’ conceptualisation of political forgiveness.

23 Watkins seems right to say that forgiveness need not bring about reconciliation, and that the notion of reconciliation is more wide-ranging than forgiveness. Moreover, he seems right to notice that the transgressor might not have repented their action (as they may view it as a justified reaction in the particular historical conditions of the past), but still be committed to liberal-democratic values in the future. Forgiveness may be possible in this situation, but not reconciliation, which would require assurance that the unrepentant transgressor, while not repudiating their action, is as committed as they are to building civic relations in the future for forgiveness to lead to reconciliation.

24 Whilst it seems to me true that one of the central goals of political forgiveness is likely to be reconciliation, I understand that this is a contestable point which not all would accept. One of the difficulties is that there is a very large literature on the concept of political reconciliation also, and this concept itself raises many definitional problems. I explore some of these definitional issues in Mookherjee (2016). Although it is not possible in the scope of the paper to discuss the relationship between these two concepts, I am very grateful to an anonymous referee for *Philosophia* for encouraging me to consider this point.

25 For Arendt, the ‘miracle’ that saves the world from its ruin is ultimately the fact of natality: ‘only the full experience of this capacity [for natality] can bestow upon human affairs faith and hope, those two essential characteristics of human existence which Greek antiquity ignored altogether, discounting the keeping of faith as a very uncommon and not too important virtue….’ (1958: 247). The suggestion in this article is that, for humans to develop the capacity for natality, they need compassion, notably the capacity to use one’s imagination and to care for the social world.
